# Treatment of steroid-refractory graft versus host disease in children

**DOI:** 10.3389/frtra.2023.1251112

**Published:** 2023-09-15

**Authors:** Francesca Gottardi, Davide Leardini, Edoardo Muratore, Francesco Baccelli, Sara Cerasi, Francesco Venturelli, Andrea Zanaroli, Tamara Belotti, Arcangelo Prete, Riccardo Masetti

**Affiliations:** ^1^ Pediatric Oncology and Hematology, IRCCS Azienda Ospedaliero-Universitaria di Bologna, Bologna, Italy; ^2^ Department of Medical and Surgical Sciences (DIMEC), University of Bologna, Bologna, Italy

**Keywords:** GvHD, steroid-refractory, children, pediatric HSCT, ruxolitinib

## Abstract

Systemic steroids are still the first-line approach in acute graft-versus-host disease (aGvHD), and the backbone of chronic GvHD management. Refractoriness to steroid represent a major cause of morbidity and non-relapse mortality after hematopoietic stem cell transplantation (HSCT). In both backgrounds, several second-line immunosuppressive agents have been tested with variable results in terms of efficacy and toxicity. Solid evidence regarding these approaches is still lacking in the pediatric setting where results are mainly derived from adult experiences. Furthermore, the number of treated patients is limited and the incidence of acute and chronic GvHD is lower, resulting in a very heterogeneous approach to this complication by pediatric hematologists. Some conventional therapies and anti-cytokine monoclonal antibodies used in the adult setting have been evaluated in children. In recent years, the increasing understanding of the biological mechanisms underpinning the pathogenesis of GvHD justified the efforts toward the adoption of targeted therapies and non-pharmacologic approaches, with higher response rates and lower immunosuppressive effects. Moreover, many questions regarding the precise timing and setting in which to integrate these new approaches remain unanswered. This Review aims to critically explore the current evidence regarding novel approaches to treat SR-GvHD in pediatric HSCT recipients.

## Introduction

Despite the improvement of transplant platforms and post-transplant immunosuppression, graft-versus-host disease (GvHD) still represents a significant complication following pediatric hematopoietic stem cell transplantation (HSCT) (1–5). The incidence of acute GvHD (aGvHD) in children is approximately 50% of any grade and 20% of grade II-IV, with certain variability based on the characteristics of HSCT (6). About half of patients with grade II-IV aGvHD do not respond to first line steroids, posing a significant challenge for clinicians (7). Chronic GvHD (cGvHD) affects between 6% and 33% of the pediatric patients, with higher incidence after peripheral blood HSCT and most important risk factor represented by previous aGvHD. While mild cGvHD can be managed with topic treatment, systemic steroid, sometimes in addition to calcineurin inhibitors (CNIs) is first-line therapy in patients with moderate/severe GvHD, but, again, only about 50% patients achieve a sustained response (8, 9).

**Figure 1:**
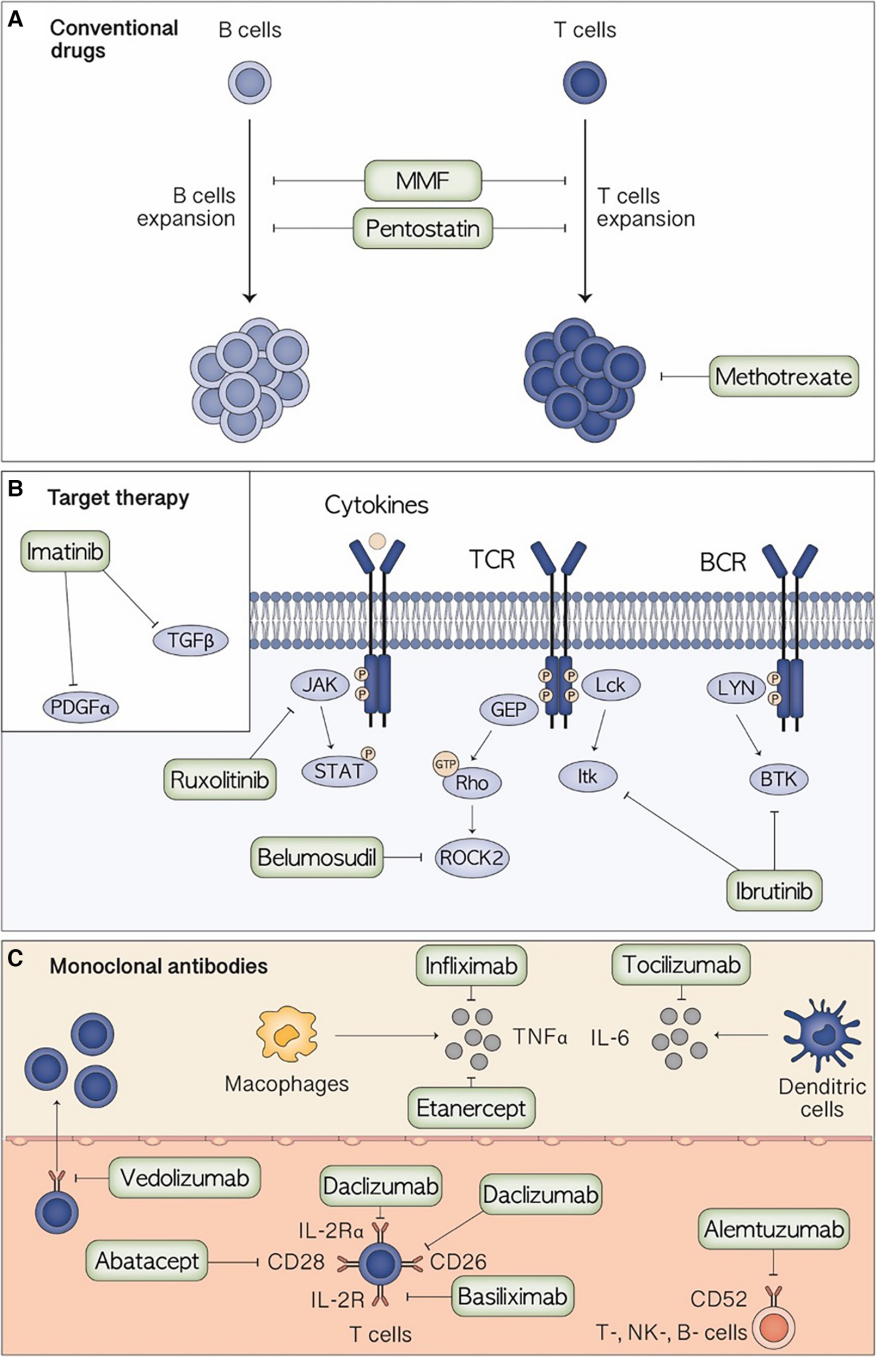
Mechanism of action of the main therapeutic agents for the treatment of pediatric steroid-resistant acute graft-versus-host disease.

Many treatments have been tested both for acute and chronic steroid-refractory (SR) GvHD based on the increasing understanding of the biological mechanisms underpinning pathogenesis. While aGvHD is caused primarily by donor T cell activation and production of pro-inflammatory cytokines, cGvHD involves both B and T cells, macrophages, and dendritic cells (DCs) converging in activating pro-fibrotic pathways (2). Introduction of therapies targeting cytokines and T cell activation pathways in aGvHD, besides both B and T cells in cGvHD, allowed to reach higher response rates with lower immunosuppressive effects (10). Indeed, among these, the JAK inhibitor ruxolitinib modified the current approach to SR acute and chronic GvHD, being the first drug FDA and EMA approved in patients aged over 12 years for these indications (11). Moreover, BTK inhibitor ibrutinib further renewed this landscape, being the first FDA-approved for refractory cGvHD fully indicated in pediatric patients. However, there are no prospective trials comparing second-line treatments or consensus guidelines for managing SR in both chronic and acute GvHD (12). Moreover, many questions regarding the precise timing and setting in which these new approaches can be integrated remain unanswered. The evaluation of different treatments for GvHD in the pediatric setting poses peculiar problems and questions. Firstly, the number of pediatric patients receiving HSCT and developing SR GvHD is lower compared to adults, resulting in a smaller cohort of treated patients with consequent delays in drug approvals. Moreover, the biology of immune cell recovery after HSCT and the GvHD development present some differences between adults and children raising questions on the different efficacy of the same drug in the two cohorts (13, 14). Lastly, children present peculiar clinical presentation of GvHD, different pharmacokinetics and unique disease that results in different GvHD presentation and response. This review aims to criticallyexplore the current evidence on novel approaches to treat SR GvHD in pediatric HSCT recipients (summarized in Figure 1) underlying the current area of research and future perspectives.

## Conventional drugs

### Methotrexate

Methotrexate (MTX) is an antifolate used at low doses for its anti-inflammatory and immunomodulatory effects: it induces a sustained suppression of T-cell activation and inhibits the production of several inflammatory cytokines that play an important role in the GvHD pathogenesis. Low-dose MTX is commonly used in GvHD prophylaxis, but evidence for its use in the treatment of GvHD is scarce, especially in the pediatric population. The main toxicities are cytopenia and nephrotoxicity. Two pediatric retrospective studies by Inagaki et al. evaluated low-dose MTX as a salvage treatment for steroid refractory and dependent acute and chronic GvHD (cGvHD), finding it tolerable and effective in reducing the dose of steroids without increasing the risk of opportunistic infections. Among 23 patients with SR aGvHD, 37% achieved complete response (CR) and 9% achieved partial response (PR) within 4 weeks without any additional agents. Resolution of aGvHD manifestations in each evaluable organ was observed in 52% with skin aGvHD and in 35% with GI aGvHD. Severe neutropenia was observed in 26% patients and thrombocytopenia in 49%. Fatal infectious complications occurred in 9% of patients. Overall response reported in pediatric cGvHD patients was 58.8% (15) (Table 1). Adult and pediatric retrospective studies were reviewed by Nassar et al. in 2014, estimating in aGvHD an overall response rate (ORR) of 69.9%, and in cGvHD 77.6%. Predictors of better response were lower grade GvHD, cutaneous involvement, and isolated organ involvement (20).

**Table 1:** Main pediatric studies on conventional drugs for the treatment of pediatric steroid-resistant acute graft-versus-host disease.

Drug, Author	Study design	No of patients (Age range)	GvHD	OR	CR	OS	Dose	Main toxicities
MTX Inagaki et al. (160)	Retrospective study	10 (4–15)	Acute	70%	50%	60%	3–10 mg/m^2^ weekly	22% severe adverse events, mostly cytopenia and elevated liver enzymes, hemorrhagic cystitis, pulmonary aspergillosis.
		17 (2–16)	Chronic	70.6%	23.5%	93.8%		
MTX Inagaki et al. (161)	Retrospective study	35 (0–18)	Acute	46%	37%	62%	10 mg/m^2^ weekly	Neutropenia 26%, thrombocytopenia 49%, Infections 49%.
MMF Inagaki et al. (16)	Retrospective study	14 (0–17)	Acute	79%	50%	85%	60 mg/kg/day (range 34–107)	CMV, ADV infections (64%) Haemorrhagic cystitis (14%), Neutropenia (7%), thrombocytopenia (7%)
MMF Kawashima et al. (22)	Retrospective study	62 (0–15)	Acute	61%	–	76%		Neutropenia (5%), infection (1.5%), thrombocytopen ia (2%), and diarrhea (1.4%)
		44 (0–15)	Chronic	36%	–	84%		
MMF + imatinib Choi et al. (17)	Phase II	13 (5–20)	Chronic	76.9%	–	84.6% at 1 year	15 to 20 mg/kg (maximum 1 g) twice daily	Increased liver enzymes (38%), renal toxicity (15%), infections (38%)
Pentostatin Bolanos-Meade et al. (18)	Phase I	23 (0.5–63)	Acute	76%	64%	26%	1–3 mg/m^2^/d for 3 days	Modest elevations of liver function tests, thrombocytopenia, lymphopenia
Pentostatin Jacobsohn et al. (19)	Phase II	51 (0.9–20.7)	Chronic	53%	26%	84% at 1 year, 60% at 3 years	4 mg/m^2^ ev every 2 weeks for 12 months	Infections, autoimmune hemolytic anemia (5%)

ADV, adenovirus; CMV, cytomegalovirus; CR, complete response; GvHD, graft-versus-host disease; MMF, mycophenolate mofetil; MTX, methotrexate; OR, overall response; OS, overall survival.

### Mycophenolate mofetil

Mycophenolate Mofetil (MMF) is the prodrug of mycophenolic acid (MPA). After oral administration MMF is rapidly absorbed and hydrolyzed to MPA, which blocks the pathway of purine synthesis in lymphocytes by selectively and reversibly inhibiting inosine monophosphate dehydrogenase, thus suppressing T and B-cells proliferation (15, 21). MMF has been used as a component of GvHD prophylaxis regimens and as a salvage treatment for refractory aGvHD and cGvHD, with limited evidence in the pediatric population. Inagaki et al. (16) retrospective study in 2015 evaluated the efficacy of MMF in a cohort of 14 pediatric patients with SR aGvHD. At 4 weeks, 50% achieved CR, up to 79% at 8 weeks. Remarkably, favorable responses were observed in most cases of gastrointestinal (GI) aGvHD. The median maximum dose of MMF given to patients was 60 mg/kg/day divided in two doses, higher than previous studies, as poor absorption of MPA was presumed due to most of the patients suffering severe gut involvement. The most common adverse reactions during treatment were opportunistic infections and cytopenia. A Japanese retrospective study in 2018 by Kawashima et al. (22) evaluated MMF in combination with other immunosuppressive therapies. Sixty-two children were treated for steroid or steroid + CNI refractory aGvHD, with an ORR of 61%. Improvement of skin involvement was observed in 65%, intestine in 27%, and liver in 8% of patients. Combined immunosuppressants were reduced in 57% and discontinued in 18% patients. In the same study, a total of 44 children received MMF for the treatment of refractory cGvHD, of which 36% had improved subjective symptoms. Concomitant immunosuppressants were reduced in 41% and discontinued in 24% patients. Major adverse events were registered in <5% of patients and were mainly neutropenia, infection, thrombocytopenia, and diarrhea. In this study MMF seems to be less toxic in children when compared with adults, as regards renal damage (22). Choi et al. in 2021 evaluated the efficacy and safety of imatinib + MMF to treat sclerotic/fibrotic type cGvHD. A total of 13 patients were enrolled, aged 5–20 years. At 1 year, 1 patient achieved CR and 8 patients achieved PR, with an ORR of 76.9%. The highest response rate was observed in the liver, namely 70%, and the lowest in the lungs and GI tract, 41.7% and 33.3%, respectively. The median steroid dose was decreased from 1.0 to 0.21 mg/kg/day. Common adverse events included elevated liver enzymes and serum creatinine levels, and fever (17) (Table 1).

### Pentostatin

Pentostatin is a nucleoside analog that irreversibly inhibits adenosine deaminase, blocking the metabolism of 2′-deoxyadenosine, with consequent accumulation of dATP that slows lymphocyte growth and causes apoptosis (18). Pentostatin has a reasonable toxicity profile, and its side effects include thrombocytopenia, neutropenia, renal toxicity, increased hepatic liver enzymes and infections (18–25). Bolanos-Meade et al. evaluated in a phase I dose escalation study pentostatin at a dose of 1–3 mg/m^2^/day for 3 days to treat 23 pediatric and adult patients with SR aGvHD. ORR was 86% and CR 64% (18). Jacobsohn et al. evaluated in a phase II prospective study 51 pediatric patients with SR cGvHD, who presented a 53% ORR. Patients with rash/lichenoid changes or sclerosis had a better response rate, 50% and 59% respectively, while none of the patients with liver or lung involvement responded to this treatment (19). The results were similar to those obtained in the adult population (26). The toxicities observed were mostly infectious, but also included 3 cases of autoimmune hemolytic anemia (19) (Table 1). More studies have been conducted in the adult population, both alone and in combination, with very variable responses, and CR achieved from 13% to 70% (23, 27, 28).

## Targeted therapies

### Imatinib

Imatinib is a tyrosine kinase inhibitor widely evaluated in cGvHD. By inhibiting both platelet-derived growth factor a (PDGFa) and transforming growth factor beta (TGFbeta) intracellular signaling. Imatinib has proved to be effective in patients with cGvHD with sclerotic/fibrotic features (17, 29). Side effects observed include transaminase elevation, renal toxicity, infections, myelosuppression, and edema because of fluid retention (17, 30). Faraci et al. retrospectively studied the use of imatinib as second-line treatment of bronchiolitis obliterans in 13 children, together with CSA, tacrolimus, and methylprednisolone pulses, with an ORR of 76.9%, CR 30.8% and PR 46.1%, and an overall survival (OS) at 4 years of 83.3%, compared to 42.6% in the group without imatinib (31). As already described above, Choi et al. treated 13 pediatric patients with SR or dependent cGvHD with fibrotic/scleroderma-like features with imatinib and MMF (17) (Table 2).

**Table 2:** Main pediatric studies on targeted therapy for the treatment of pediatric steroid-resistant acute graft-versus-host disease.

Author	Study design	No of patients (age range)	GvHD	OR	CR	OS	Dose	Main toxicities
Imatinib + CNI and mPDN pulses Faraci et al. (31)	Imatinib + CNI and mPDN pulses Retrospective	13 (0–18)	Chronic (BOS)	76.9%	30.8%	83.3% at 4 years	100–200 mg/m^2^/die	Peripheral generalized fluid retention (15%)
Imatinib + MMF Choi et al. (17)	Phase II	13 (5–20)	Chronic	76.9% at 1 year	7% at 1 year	84.5% at 1 year	260 mg/m^2^/die (max 400)	AST/ALT elevation (grade 3: 30.8%), renal toxicity (grade 2: 15.4%), infections (46.2%), pain (7%), decreased bone mineral density (7.7%
Ibrutinib Carpenter et al. (32)	Phase 1–2 iMAGINE trial (NCT03790332)	59 (1–22)	Chronic	61% (SR-group)	4% (SR-group)	95% at 1 year	240 mg/m^2^/day (up to 420 mg)	Grade >3: Pyrexia (31%), diarrhea (25%)
Belumosudil Cutler et al. (33)	Phase 2 randomized multicenter registration ROCKstar study NCT03640481.	132 (≥12 year)	Chronic	76% best ORR	5% at 1 year	89% at 2 years	200 mg daily or 200 mg twice daily	Fatigue, diarrhea, nausea, elevated liver function tests and respiratory tract infections. Drug-related severe adverse events in 5%, 12% stopped treatment because of possible drug-related toxicities
Ruxolitinib Zeiser et al. (49)	Phase 3 randomized trial multicentric REACH2 NCT02913261	154 (≥12 year)/5 (12–18 year	Acute	62.3% ORR at day 28; 39.6% at day 56	34.4% CR at day 28; 26.6% CR at day 56	49% at 1 year	10 mg twice daily (dose modifications for adverse events); tapering after day56 if response	Thrombocytopenia (33%), anemia (30%), CMV infection (26%), peripheral edema (18%), Neutropenia (16%) Sepsis (7%), EBV infection (6%) Serious adverse events up to day 28 (38%); treatment discon- tinuation (11%)
Ruxolitinib Zeiser et al. (50)	Phase 3 randomized trial multicentric REACH3 NCT03112603	165 (≥12 year)/4 (12–18 year)	Chronic	49.7% ORR at week 24	6.7% CR at week 24	81.4% at 1 year	10 mg twice daily for at least 6 cycles (28 days/ cycle) unless unacceptable side effects or progression of cGVHD	Anemia (29.1%), thrombocytoepnia (21.2%), liver enzymes increased (15.2%), creatinine increased (13.9%). Serious adverse events up to week 24 (33.3%); treatment discontinuation (16.4%)
Ruxolitinib Khandelwal et al. (34)	Retrospective monocentric	11 (1.6–16.5)	Acute	45% ORR at 4 weeks	1/11	7/13 alive at 401 days	2.5 mg twice daily (<25 kg)/5 mg twice daily (>25 kg); if tolerated dose escalation until a maximum of 10 mg twice daily	Liver enzymes elevation, Neutropenia, Thrombocytopenia, Infections (all successfully treated with antimicrobial therapy)
Ruxolitinib Gonzales Vicent et al. (52)	Retrospective monocentric	22 (0.5–18)	Acute (13) and chronic (9)	Acute: 77% ORR (best response); chronic: ORR 89% (best response)	Acute: 31% CR (best response); chronic: CR22% (best response)	62% at 716 days	2.5 mg once daily (infants)/2.5 mg twice daily (<25 kg)/5 mg twice daily (>25 kg)/10 mg twice daily (>12 years).	Mild thrombocytopenia, infections (54%).
Ruxolitinib Uygun et al. (35)	Retrospective monocentric	29 (0.3–17.5)	Acute (13) and chronic (16)	Acute: 85% ORR; chronic: 81% ORR (best response)	**Acute**: CR 70%; chronic: CR 6%	N/A	2.5 mg twice daily (<15 kg)/5 mg twice daily (>15 kg), dose increased until a maximum of 10 mg twice daily. Dose reduction if azole treatment.	Cytopenia, CMV infection
Ruxolitinib Laisne et al. (36)	Retrospective multicentric	29 (0.6–14.5)	Acute	ORR 89% (best response)	CR 65.5% (best response)	23/29 pts alive at a median follow-up of 685 days (177–1042 days) after the HSCT	Median initial dose 12.6 mg/m^2^/day (6.3–28.7 mg/m^2^/d)	Viral infections (41.4%), thrombocytopenia (10.3%)
Ruxolitinib Moiseev et al. (37)	Prospective single-center open-label study (NCT0 2997280)	34 (pediatric and adults)	Acute (17) and chronic (17)	ORR 75% (best response)	CR 63% (best response)	Acute 59%; chronic: 85% at median follow-up 28 months [23–47 months]	10 mg BID (adults and children >40 kg)//0.15 mg/kg BID (children < 40 kg); dose modification if adverse events	Acute: anemia (86%), neutropenia (41%), thrombocytopenia (77%), CMV infection (59%); chronic: cytopenia (15%)

AST/ALT, aspartate aminotransferase/alanine aminotransferase; CMV, cytomegalovirus; CNI, calcineurin inhibitor; CR, complete response; EBV, epstein-barr virus; GvHD, graft-versus-host disease; MMF, mycophenolate mofetil; mPDN, methylprednisolone; OR, overall response; ORR, overall response rate; OS, overall survival.

### Ruxolitinib

Ruxolitinib is an oral selective Janus kinase (JAK) 1 and 2 inhibitor, first approved for the treatment of myelofibrosis and polycythemia vera in adults (38). The JAK 1/2 kinases are involved in cellular proliferation and activation, via the activation of STAT signaling (38). This pathway is critical in T-cell function (39) and has been studied as a potential target in immune disorders (40–42), being also involved in GvHD pathogenesis (43). Ruxolitinib was demonstrated to control clinical features of GvHD (44) and demonstrated to preserve the graft vs. leukemia (GvL) effect in preclinical models (45). Subsequently, its use has been tested in adult patients with acute and chronic SR GvHD resulting to be both effective and safe (46, 47). The prospective trial REACH1 (NCT02953678), an open-label, single-arm, multicenter trial of ruxolitinib in patients 12 years and older with SR and steroid-dependent aGvHD showed an ORR at any time of 73,2% with CR of 56.3% (11, 48). Two multicenter, randomized, open-label, phase 3 trials, REACH2 (NCT02913261) and REACH3 (NCT03774082), confirmed the efficacy of ruxolitinib in acute and chronic SR GvHD, respectively. OR resulted higher than best available therapies, namely 62% vs. 39% for 28 days aGvHD response, and 49% vs. 26% for cGvHD response after 24 weeks. Failure-free survival was also higher in the ruxolitinib group (11, 49). Based on these results, ruxolitinib was thus approved for the treatment of SR acute and chronic GvHD in patients >12 years by the FDA in 2019 and subsequently by EMA (50). Pediatric studies on the use of ruxolitinib in GvHD have been increasingly reported worldwide in recent years. In the under-12-years age group, ruxolitinib has been used off-label for SR GvHD. Eleven studies evaluated children with aGvHD treated with ruxolitinib were available (34–57). Results of most relevant pediatric studies are summarized in Table 2. ORR to ruxolitinib varies from 45% to 100% and CR from 9% to 67,5%. Treatment failure (TF) was reported in a range of 17%–36% and non-response (NR) varies from 0% to 25%. The NCT02997280 prospective study by Moiseev et al. showed in multivariate analysis a lower response rate in grade III-IV, liver and grade IV GI aGvHD, while no transplantation or donor characteristics were associated with response (37). Among 29 children in the report by Laisne et al, no association of baseline characteristics, GvHD characteristics or previous immunosuppressive therapies with response to ruxolitinib was found (36). Nine studies described treatment with ruxolitinib in children with SR cGvHD (10, 35, 51, 53–58). ORR was variable from 50% to 100%, with CR from 0 to 28%. In the prospective study NCT02997280, none of the transplantation and donor characteristics were predictive for response (37). Generally favorable response rates were reported for lung GvHD/bronchiolitis obliterans (50%–90%) (10). Studies describing the use of ruxolitinib in children, generally show a good toxicity profile. Cytopenia represented the most frequent complication, mainly neutropenia and thrombocytopenia, ranging from 0 to 69% and 0 to 67%, respectively, but generally of low-moderate grade. Liver toxicity was also frequent but rarely was cause of the treatment discontinuation. Infections were also common, including bacterial, viral, and fungal infections, with few severe cases reported, including sepsis and adenovirus infections. Notably, CMV reactivation was common during ruxolitinib administration, but no CMV-related death was documented. Importantly, REACH4 (NCT03491215), a phase 1/2 open-label, single-arm, multicenter clinical trial is ongoing to evaluate addition of ruxolitinib to steroid therapy in pediatric patients with grade II-IV treatment-naïve or SR aGvHD. In a preliminary analysis on 32 patients with SR aGvHD, a OR at day 28 was 90.6% and OR at day56 was 68.8% (59).

### Ibrutinib

Ibrutinib is a selective and irreversible Bruton's Tyrosine Kinase (BTK) inhibitor. BTK is predominantly expressed in B cells and its activation is critical for B-cell survival, proliferation, and migration. Ibrutinib has been originally used in B-cell malignancies, as it arrests cell growth and induces apoptosis. Thus, ibrutinib is FDA and EMA approved for chronic lymphocytic leukemia and relapsed/refractory mantle cell lymphoma (60) In addition to inhibiting BTK, ibrutinib is an irreversible inhibitor of Interleukin-2 inducible Tyrosine Kinase (ITK), involved in T-cell receptor signaling and activation, cytokine release, and proliferation (61). Ibrutinib was identified as a potential treatment for cGvHD, characterized by chronic inflammatory responses driven by alloreactive T-cells, pro-fibrotic pathways, and B-cells produced anti-host antibodies (62). A phase 2 clinical trial by Standford University in 2017 culminated in the FDA approval of ibrutinib as second line therapy for SR cGvHD in adults (63). A few years later, in 2022, ibrutinib received its approval in the US for its use in pediatric patients of 1 year and older with cGvHD after the failure of one or more lines of systemic therapy. Ibrutinib thus represents the first ever approved treatment in this specific group of patients (32, 64). Efficacy as a treatment for moderate or severe cGvHD was demonstrated in 59 patients aged 1–22 years after the failure of one or more lines of systemic therapy and in those who were newly diagnosed and previously untreated (64). In the overall population, a sustained response for ≥20 weeks was seen in 61% of those who had achieved a partial or complete response. The 12- and 18-month OS estimates in the overall population were 95% and 91%, respectively. Improvement occurred in multiple organ systems and responses lasted ≥5 months in half of the patients. Response to ibrutinib permitted reduction of glucocorticoid dose to ≤0.15 mg/kg/day in nearly two-thirds and was associated with improved quality of life. The most common adverse reactions with ibrutinib in the overall population were pyrexia (31%) and diarrhea (25%) (Table 2). Gagliardi et al. recently reported a small experience of combination therapy of ibrutinib with ruxolitinib for steroid refractory cGvHD in two pediatric patients and found this combination to be well tolerated with no significant adverse events for neither patient had to discontinue these drugs (65).

### Belumosudil

Targeted approaches that directly address inflammation and fibrosis associated with cGvHD have been developed. The rho-associated coiled-coil-containing protein kinase-2 (ROCK2) promotes the production of the proinflammatory cytokines IL-21 and IL-17, downregulates STAT5 inhibiting Treg differentiation and upregulates profibrotic gene expression (66, 67). The oral Selective ROCK2 inhibitor Belumosudil, previously known as KD025, exerts multiple effects *in vitro* and in preclinical models by inhibiting IL-21, IL-17, and IFN*γ* secretion, reducing Th17 and follicular helper cells via downregulation of STAT3, and enhancing regulatory T cells via upregulation of STAT5. It also seems to inhibit fibroblast proliferation and collagen production and reduce profibrotic M2 macrophage differentiation (68, 69). In the murine model, ROCK2 inhibition was effective in ameliorating sclerodermatous cGvHD and bronchiolitis obliterans by modulating the immune system and reducing lung and skin fibrosis (69). These promising data lead to the design of two phase 2 trials. In the phase 2 dose-finding trial including only patients older than 18 years, belumosudil treatment with 200 mg daily or twice daily resulted in a OR of 65% and 69% respectively, and it was associated with significant corticosteroid dose reduction (70). The ROCKstar phase 2 randomized multicenter registration study included patients of 12 years and older to evaluate belumosudil 200 mg daily or twice daily in patients non responder to 2 to 5 prior lines of therapy. The primary endpoint was best ORR. The trial enrolled 132 subjects, with a median follow-up of 14 months. belumosudil 200 mg daily or twice daily resulted in a the best ORR of 74% and 77% respectively, with a median duration of response of 54 weeks. Response rates were high in all affected organs and even after failure of ibrutinib and/or ruxolitinib. Patient-reported symptom reduction was also reported in 59% and 62%, respectively. Belumosudil was well tolerated in these heavily pretreated subjects, with 44% of patients continuing treatment for more than 1 year. Toxicities mostly consisted of fatigue, diarrhea, nausea, elevated liver function tests and respiratory tract infections. Drug-related severe adverse events occurred in 5% of subjects, and 12% discontinued belumosudil because of possible drug-related toxicities (33). This trial led to FDA approval for adult or pediatric patients 12 years and older with chronic GvHD after the failure of at least 2 prior lines of systemic therapy, with a starting dose of 200 mg orally once daily (67). To date, belumosudil has not been approved by EMA yet. A combined analysis from 2 prospective trials outlined best ORR for lung cGvHD of 32%, with CR of 15%. Response rates were inversely proportional to baseline *National Institute of Health* NIH GvHD lung score at enrollment (71). Interestingly, the introduction of belumosudil in the care of cGvHD has been associated with substantial cost savings in the US, mainly due to reduced adverse events and less healthcare resource utilization (72).

## Monoclonal antibodies

### Basiliximab and daclizumab

Basiliximab, a chimeric monoclonal antibody, binds to the interleukin-2 receptor on activated cytotoxic T-cells, inhibiting lymphocyte proliferation, and reducing tissue damage, and has been considered as a treatment option for SR aGvHD in adults (73–77). Studies in the pediatric population are lacking. To date, studies including both children and adults showed good results in retrospective cohorts (75–78). A pediatric only study was carried on by Tang et al. the setting of SR-aGvHD in haploidentical HSCT. The authors retrospectively reviewed 100 patients with an ORR at day 28 of 85%, and CR in 74% of cases. OS was significantly higher in responders compared to non-responders, 81% vs. 47%. Basiliximab was well tolerated without any infusion-related side effects. CMV reactivation was the most common infection during treatment, occurring in 53% of patients, while 11% and 7% developed bacterial and fungal infections, respectively. These rates were comparable to the ones of adults (78). Daclizumab is a humanized monoclonal antibody directed vs. IL-2Ralpha. It has been tested in a prospective study in children with SR GI aGvHD with a ORR of 85%. Treatment was well tolerated, but infections were common. Four patients subsequently developed cGvHD (79). Miano et al. described 13 pediatric patients treated with daclizumab for SR aGvHD with CR 46% and ORR 92%. Even in this study, 50% of patients developed cGvHD (80) (Table 3).

**Table 3:** Main pediatric studies on anti-cytokines and monoclonal antibodies for the treatment of pediatric steroid-resistant acute graft-versus-host disease.

Study	Type of study	No of patients (Age range)		Type of GvHD	OR	CR	OS	Dose	Side effects
Basiliximab Tang et al. (78)		Retrospective study	100 (0–18 year)	Acute after Haplo-HSCT	85% at day 28	74% at day 28	81,3% at 3 years	20 mg > 35 kg and 10 mg < 35 kg. Day 1–3 than weekly	CMV infections (53%), bacterial, fungal infections
Daclizumab Hamidieh et al.		Prospective study	13	Acute GI	11/13	10/13	10/13	1 mg/kg intravenously repeated 10–14-day interval maximum 5 doses	Infections
Daclizumab Miano et al. (86)		Retrospective study	13	Acute	92%	46%	46%	1 mg/kg i.v. days +1, +4, +8, +15 and +22	Infections in 12 patients
Tocilizumab Bhatt et al.		Retrospective series	4	Acute	67%	50%	1 death for IFI	8 mg/kg every 3–4 weeks	Infections
			2	Chronic	50%	50%			
Tocilizumab Beebe et al.		Retrospective series	5	Chronic	100%	1/5		8 mg/kg intravenously every 3 weeks	Sinusitis, flu
Infliximab Sleight et al. (100)		Retrospective study	24 (0–18 year)	Acute	82%	12/22 (54%)	46% 12 months, 13% 36 months;	10 mg/kg i.v. once a week for a median of eight doses	Infections (77% bacterial, 32% viral 13.6% IFI)
Infliximab Yang et al. (162)		Retrospective study	10	Acute	10/10 (Reported 5/10 cGvHD)	8/10	40%	10 mg/kg infliximab weekly for 3–4 doses	Infections
Etanercept Faraci et al. (81)		Prospective study	25	Acute	68%	56%	59.1% (76.5% in responding vs 16.7%)	0.4 mg/kg s.c. twice weekly for 8 weeks (16 doses total)	Bacteremia 36%, viral reactivations 76%, invasive mycoses 20%
Alemtuzumab Khandelwal, (115)		Retrospective study	19	Acute	73%	47%	52% 2 years	Median dose of 0.9 mg/kg (range 0.3–2 mg/kg) divided over 2–6 days.	Bacteremia 47%, fungal infections 21%, adenovirus 52%, EBV 36%, CMV viremia 36%
Begelomab Bacigalupo et al. (82)		Compassionate use study with 7 ped. patients separately described for response	7 (3–20)	Acute	6/7 (86%)	–	Not available for pediatric patients	3 mg/m^2^/day for 5 days, 6 additional doses	Not specifically reported in pediatric patients, in the whole study mainly diarrhea and viral infections
Vedolizumab Ibrahimova et al.		Case series	4	Gut aGvHD	3/4 (75%)	1/4 (25%)	50% at 6 months	150–300 mg weekly	None
Vedolizumab Isshiki et al.		Case series	3	Gut aGvHD	3/3 (100%)	2/3 (67%)	100%	177 mg/m^2^/dose weekly	None
Vedolizumab Rosa et al.		Case series	3	Gut aGvHD	1/3 (33%)	1/3 (33%)	33% at 1 year	300 mg weekly	None
Vedolizumab Fukuta et al.		Case report	1	gut aGvHD	1/1 (100%)	1/1 (100%)	100% at 5 years	6 mg/kg	None
Vedolizumab Aldouby Bier et al.		Case series	13	Gut aGvHD	10/13 (77.0%)	8/13 (61.5%)	76.9% at 10.5 months	100 mg for <10 kg, 150 mg for 10–25 kg, 300 mg for >25 kg	Infections
Abatacept (+Basiliximab and Etarnecept) Rani Jaiswal et al. (83)		Observational monocentric study	5 (2–20)	Acute (hyperacute)	100% at day 29; 40% at day 56	2/6 sustained CR	2/6 alive (responder)	10 mg/kg on days 1, 8 and 22	No acute toxicity (no infections during the first 6 weeks of treatment)

aGvHD, acute graft-versus-host disease; cGvHD, chronic graft-versus-host disease; CMV, cytomegalovirus; CR, complete response; GvHD, graft-versus-host disease; HSCT, hematopoietic stem cell transplantation; IFI, invasive fungal infections; OR, overall response; OS, overall survival.

### Infliximab and etanercept

TNF*α* is a key cytokine in the inflammatory cascade of GvHD. Secreted alongside interleukin-1 by macrophages residing in the host's mucosae, TNF-α triggers the proliferation of donor T-cells and stimulates the secretion of interleukin-2 and interferon-α, resulting in the amplification of T-lymphocytes and mononuclear phagocyte responses. The damage inflicted upon the intestinal mucosa facilitates the translocation of lipopolysaccharides from the normal bowel flora and other immune-stimulatory molecules from the intestinal lumen into the bloodstream. This, in turn, propagates the characteristic cytokine storm observed in aGvHD (84). Furthermore, TNFα and soluble TNF*α* receptor I and II have been shown to be correlated with aGvHD severity (85, 86). Thus, the use of TNFα inhibitors to manage GvHD have been suggested in the primary prophylaxis (87, 88), in first-line treatment (89, 90) and in SR aGvHD.

Infliximab is a chimeric human-murine IgG1κ monoclonal antibody that binds to the soluble and transmembrane isoforms of TNF-α and inhibits their binding with the cellular receptors (91). Its potential role in the treatment of SR GvHD has been explored since the early 2000s (92, 93). In the pediatric setting, Sleight et al. reported that weekly infusions of infliximab at the dose of 10 mg/kg are effective for children with both acute and chronic refractory GvHD, especially for children with skin and gut involvement (94) with an ORR of 82%. Nevertheless, long-term outcome was less satisfying, with common recurrence of GvHD upon discontinuation of infliximab and a significant number of infections within 100 days of the final dose, up to 77% bacterial, 32% viral and 13.6% probable proven invasive fungal infections (94). In Yang et al. experience, 10 pediatric patients with SR aGvHD were treated with a CR rate of 80%. However, infections were reported in all patients, 5 viral infections, 2 atypical mycobacterial infections. 3 invasive pulmonary aspergillosis, and 6 patients had multiple infections. Moreover, 50% developed cGvHD, 60% died during follow-up. A recent multicentric study on treatment of pediatric SR aGvHD reported that infliximab was the second most utilized therapy, in 30%, but in half of the cases was utilized in combinations with other agents, such as vedolizumab, basiliximab, etanercept, tacrolimus and/or ruxolitinib (95). Finally, infliximab-daclizumab combination was used to treat acute and chronic liver and GI SR GvHD in two children, with complete response in both (96). The use of infliximab in chronic GvHD has been less explored, with no relevant studies particularly in pediatric patients.

Etanercept is a recombinant human soluble dimeric TNF*α* receptor fusion protein that binds and inactivate TNF*α*. Most experiences in the use of Etanercept for GvHD treatment were gathered from cohort of adults (97, 98), with few reports on children alone. In adults, responses in gut SR aGvHD have been described, but appear to be associated with poor long-term survival even in responding patients (99). Notably, Faraci et al. prospectively evaluated use of etanercept in 25 children with SR aGvHD, concluding an ORR of 68% (81). It must be mentioned that clinically significant infectious complications requiring systemic treatment occurred in 68% of patients, mainly bacterial and viral reactivations. OS was 77% in responders and 17% in non-responders (Table 3).

### Tocilizumab

Tocilizumab is a humanized monoclonal antibody against the inflammatory cytokine IL-6. Since GvHD is characterized by dendritic cell driven IL6 dysregulation after HSCT (100), tocilizumab has been proposed for treatment of both acute and chronic GvHD. In adults, a CR rate of 63% was reported in patients affected by SR low GI aGvHD (101) and an OR rate of 70% was reported in patients with extensive cGvHD (102). In a retrospective pediatric series, tocilizumab was administered to 6 patients with SR aGvHD and 2 with cGvHD every 3 to 4 weeks. Infections were the primary adverse events associated with tocilizumab administration. OR was 67% in aGvHD and ½ patient with cGvHD had a significant response to therapy, whereas the second had stabilization of disease that allowed for a modest reduction in immune suppressive medications. Beebe et al. reported 5 children and young adults with cGvHD treated with tocilizumab. All patients reported subjective improvement of cGvHD, reducing use of additional immunosuppression by >50%, and one patient discontinued steroids after 5 years of dependency. Treatment was affected by mild infections. Interestingly, four patients had normal IL-6 levels prior to starting treatment (103, 104) (Table 3).

### Alemtuzumab

Alemtuzumab (Campath-1H) is a humanized IgG1 monoclonal antibody that binds cells expressing the CD52 antigen, such as T-, NK-, and B-lymphocytes as well as a proportion of monocytes and dendritic cells (105). The effect produces an *in vivo* lymphocyte depletion; thus, this molecule has been commonly adopted in HSCT conditioning to promote engraftment and prevent GvHD. Even if its use is less frequent in pediatric HSCT as alternative to serotherapy in aGvHD prohylaxis, it has been used especially in reduce-intensity conditioning (RIC) and nonmalignant disease setting. Successful use of Alemtuzumab for SR aGvHD has been reported from case series including adult patients (106–108). Generally, responses were remarkable, but virus reactivation and bacterial infections were common. Also, subsequent development of chronic GvHD was observed frequently. In pediatric patients, a retrospective study reviewed 19 patients with SR aGvHD who received alemtuzumab with 47% CR and an ORR of 73%. Infectious complications were reported in OS was significantly higher in patients treated (52% vs. 0% at 2 years) (108) (Table 2).

### Begelomab

Begelomab is a murine IgG2B monoclonal antibody directed against the CD26 surface antigen, which promotes T cell migration. Accumulation of CD26+ T cells has been proven in GVHD target organs (109). Bacigalupo et al. reported on a cohort of 69 adult patients treated with begelomab with different treatment schedules in combination with cyclosporin and steroids for steroid refractory acute GvHD. In both the prospective and compassionate groups, responses to treatment at day 28 were 75% and 61%, respectively. Responses for grade III GvHD were recorded in 83% and 73% of patients, while responses for grade IV GvHD were recorded in 66% and 56% of patients in the two groups, respectively. Interestingly, favorable responses were reported for skin, liver, and gut stage III–IV GvHD, with 64%, 56%, 68% of responses respectively. Notably, in a small subgroup of patients under 20 years of age, 87% showed response to treatment, compared to 57% and 68% in patients aged 21–40 and over 40 respectively (82). While the use of begelomab for aGvHD shows promising results, there is no clinical trial investigating the effect of begelomab in patients with cGvHD. However, preclinical models have shown that CD26 may play a role in the development of pulmonary cGvHD, and that treating human umbilical cord blood transplanted mice with the fusion protein caveolin-1-Ig, prevents the development of pulmonary cGvHD in these mice (109) (Table 3).

### Vedolizumab

Vedolizumab, a monoclonal antibody targeting α4β7 integrin, has emerged as a potential therapeutic option for the management of pediatric SR GvHD. Its mechanism of action involves inhibiting of the trafficking of gut-homing lymphocytes to the gastrointestinal tract and it was first tested in ulcerative colitis and Crohn's disease (110). In the HSCT context, preclinical studies demonstrated that loss of α4β7 integrin may prevent intestinal GvHD (111) and, based on these results, was tested in patients. In adults, a Phase II study (NCT02993783) revealed low efficacy and a poor response rate, leading to the premature discontinuation of the study (112). However, other reports have shown more positive outcomes, with response rates around 27% (113). Limited evidence exists for the use of vedolizumab in children, which mainly consists of retrospective case reports or case series. Ibrahimova et al. reported four patients with SR grade III-IV gut aGvHD, out of which only 1 achieved a complete response (114). Isshiki et al. described 3 pediatric patients with grade II-IV gut acute gut GvHD who were treated with vedolizumab. Two of these patients experienced a complete response (115). Rosa et al. reported 3 pediatric patients with oncological diseases and grade IV gut aGvHD, of whom only one achieved GvHD remission and Fukuta et al. reported 1 patient with a clinical response to vedolizumab (116, 117). Aldouby Bier reported on 13 pediatric patients with SR gut aGvHD treated with vedolizumab, among whom 8 presented a clinical recovery and 2 had ongoing chronic colitis. Interestingly, these patients experienced several infectious episodes primarily associated with intestinal bacteria, which raises some potential safety concerns (Table 3).

### Abatacept

Abatacept or cytotoxic T-cell-lymphocyte-4 (CTLA4)-immunoglobulin, is a fusion protein between the extracellular domain of human CTLA4 and a modified Fc region of human IgG. It inhibits the co-stimulation of T-cells by blocking the interaction between CD28 and CD80/CD86 on antigen-presenting cells (118). Abatacept has been initially approved for the treatment of rheumatoid arthritis (119). The drug resulted able to prevent GvHD in preclinical models (120). A Phase 2 clinical trial demonstrated the safety and efficacy of abatacept in preventing aGvHD (121). Other studies have also showed the feasibility of this approach in different pediatric settings (122, 123). Abatacept has been FDA-approved for aGvHD prophylaxis (combined with a calcineurin inhibitor and MTX) in patients undergoing unrelated donor HSCT. Reports about the use of Abatacept for treatment of GvHD are limited, particularly in children. In a report on children who have received a haploidentical HSCT with post-transplant cyclophosphamide based GvHD prophylaxis, abatacept was added to etarnecept and basiliximab in 5 children with hyperacute SR GvHD reporting an overall response at day 29 and day 56 of 100% and 40%. Response was higher compared to patients treated with a “standard” protocol including anti-thymocyte globulins combined with etarnecept and basiliximab, suggesting that T costimulation blockade combined with anticytokine agents can ameliorate the response in this particularly high-risk category of patients (83). Abatacept has been described as salvage therapy in cGvHD in a recent retrospective report on 15 patients with a wide range of age (5–70 years). Abatacept resulted a promising option for cGvHD with a best ORR of 40%, particularly high in patients with bronchiolitis obliterans in which reached 89%. Unfortunately, specific data about pediatric patients treated in this study are not available (124).

## Nonpharmacological treatments

### Extra-corporeal photopheresis

Extra-corporeal photopheresis (ECP) therapy is based on exposition of peripheral blood mononuclear cells to photoactivated 8-methoxypsoralen, followed by reinfusion of treated cells, which exert an immunomodulatory effect. Non-exposed antigen presenting cells, can phagocyte treated cells, with consequent secretion of anti-inflammatory cytokines and chemokines, modulation of T cells toward a Th2 phenotype, and Treg regeneration (125). This therapy has been widely explored in SR GvHD, as generally considered a safe and effective strategy, with limited evidence of increased infectious risk in the post HSCT setting. Main limitations are related to logistic feasibility and vascular accesses, which requires patients to be sufficiently stable (126, 127). Moreover, most centers require at least 1 × 109/L WBC in the peripheral blood before initiating the ECP therapy, limiting access to patients with cytopenia, especially in aGvHD setting (128).

The earliest evidence of efficacy of ECP in pediatric aGvHD and cGvHD was reported in 2003 by Messina et al, 33 patients with aGvHD involving skin, liver and gut had CR in 76%, 60% and 75% respectively, and of 44 children with cGvHD, 15 (44%) showed a complete response and 10 (29%) a significant improvement after treatment (129). ECP feasibility and efficacy was subsequently evaluated in various retrospective studies, but the majority involved adult patients, and as a whole, there were no major changes in the technique (125, 130).

More recently, a meta-analysis of prospective clinical trials evaluating ECP in patients with SR aGvHD reported an ORR of 71% each (131). In pediatric population, a retrospective study of 15 patients was reported from Winther-Jørgensen et al. in 2019. In aGvHD group, 67% had ORR at day 28 up to 89% at last session. Among cGvHD patients, 67% reported a PR. Only few procedure-related mild side effects were registered, even in patients with low body weight. The most frequent cause of shortened or canceled ECP treatment was difficulties with vascular accesses (132). In 2022 a retrospective study evaluated a total of 701 ECP sessions performed on 33 children. In total, 97% of the sessions could be performed, while in 8% an incident was detected, most of them mild and related to catheter dysfunction. ORR was 70% with a median time to best response of 2.8 months (133) (Table 4). It has to be mentioned that, in recent years, the potential complementary mechanisms of action of ruxolitinib and ECP has been investigated on both acute and chronic GvHD. Data have been described in adult cohorts, specifically in 18 patients with severe lower GI SR aGvHD, with ORR of 55%. During treatment with ruxolitinib and ECP, an increased level of regulatory T cells could be observed elucidating direct effects of this treatment on immune response (140). In retrospective analysis of 23 patients treated with ruxolitinib-ECP combination as salvage therapy for SR cGvHD, ORR was 74% including 9% CR (141). In both studies main toxicities were non-severe cytopenia and CMV reactivations (139, 141). Data about combination in children lacks.

**Table 4:** Main pediatric studies on nonpharmacological interventions for the treatment of pediatric steroid-resistant acute graft-versus-host disease.

Study	Type of study	No of patients (Age range)	Type of GvHD	OR	CR	OS	Dose	Side effecs
ECP Signe Winther-Jørgensen (121)	Retrospective	9 (6–14)	Acute	8/9 (88,9%)	0	100%	aGVHD: weekly cycles tapered in 3–6 months; cGVHD: one cycle every second week for 3–6 months tapered to monthly	2/15 CVC related sepsis, Mild symptoms
		6 (6–14)	Chronic	4/6 (66,7%)	0	100%		
ECP Asensi Cantò (133)	Retrospective	29	Acute	66%	52%	58%	2/weeks then tapered	Mostly related to vascular accesses, 1 case of lethal septic shock
		9	Chronic	50%	20%		1/week then tapered	
Haploidentical BM MSC Le Blanc et al. (134)	Case report	1 (9y)	Acute	1/1	1/1 (after 2nd infusion)	Alive at 1 year	1st: 2 × 106 cells/kg 2nd: 1 × 106 cells/kg	Any reported
Third party allogeneic MSC Müller et al. (135)	Case report	3	Chronic	1/3				Any reported
HLA-identical, haploidentical, and third-party HLA-mismatched MSC Le Blanc et al. (136)	Multicenter non-randomized study	25/55	Acute	80%		53% in responders vs 16% in non-responders (whole cohort, unknown in children)	Median dose of 1·4 × 10⁶ (range 0·4–9 × 10⁶) cells per/kg	Any reported but among responders, 9 died from infections (whole cohort)
Platelet-lysate-expanded MSC Lucchini et al. (137)	Retrospective	6	Acute	71.4%	23.8%	8/11 median follow-up of 8 months	Median 1.2 × 10 (6)/kg (range: 0.7–3.7 × 10 (6)/kg)	No acute and late side effects reported at a median follow-up of 8 months
		5	Chronic	3/5	1/5			
Allogeneic-MSC Ball et al. (138)	Retrospective study	37	Acute	84%	65%	Median follow-up of 2·9 years 19/37 alive	Median 2 × 106/Kg infusions, median 2	Any infusion related, not reported increase of infections
MSC (remestemcel-L) Kutzberg et al. (154)	Retrospective, NCT00759018	241	Acute	65,1%	14,1%	66% at 100 days (82% responder vs. 39% non-responder	8 bi-weekly i.v. 2 × 106 hMSCs/kg for 4 weeks, +4 additional weekly infusions after day +28 for PR	No of infusion-related toxicities or ectopic tissue formation. Most frequent SAEs were infections (24%) and respiratory disorders (16%).
MSC (remestemcel-L) Kebriaei et al. (153)	Multicenter, randomized, phase III	27/260 (14 treated)	Acute	64.3%	64.3%	34% (whole cohort, unknown in children)	8 iv infusions over 4 weeks, in addition to second-line therapy	Peripheral edema (35%), abdominal pain (22%), and thrombocytopenia (22%); Any grade infections 88% treated vs 81% placebo
MSC (remestemcel-L) Kurtzberg et al. (153)	Phase III, prospective, single-arm, multicenter study NCT02336230	54	Acute	79,4%	29.6% day28 to 44,4% day 100	78.9% vs. 43.8% non-responders at day 180	2 × 106 cells/kg twice weekly for 4 weeks	Adverse events (17%), 10 non serious including cytopenia, CMV infection, nausea, vomiting, pyrexia, allergic transfusion reaction, and hypotension. serious: skin GVHD, adenovirus, BK, hemolytic uremic syndrome, hypermetabolism, and somnolence
FMT Zhong et al. (163)	Case report	1 (5 years)	Acute	1/1	1/1	Alive at 3 months	100 ml	No adverse event reported
FMT Merli et al. (158)	Case report	1 (5 years)	Acute	1/1	CR after multiple infusions, transient	Alive after 5 years	12 ml/Kg	Nausea (grade 2) abdominal pain (grade 2) and low-grade fever (grade 1).

aGvHD, acute graft-versus-host disease; cGvHD, chronic graft-versus-host disease; CR, complete response; CVC, central venous catheter; FMT, fecal microbiota transplantation; GvHD, graft-versus-host disease; MSC, mesenchymal stem cells; OR, overall response; OS, overall survival.

### Mesenchymal stromal cells

Mesenchymal stromal cells (MSCs) can be isolated from various tissues, such as bone marrow, adipose tissue, umbilical cord, Wharton's jelly, placenta tissue, and decidua. They have shown activity in the treatment of GvHD due to their immunomodulatory properties on T, B and NK cells and capability of influence the differentiation and function of dendritic cells. MSCs release anti-inflammatory molecules, such as IL-10 and TGF-beta, dampening the inflammatory response associated with GvHD. MSC migrate to injured tissues and promote tissue repair and regeneration through their differentiation potential. In the crosstalk with immune system, they exert paracrine activity involving secretion of hormones and peptides, transfer of mitochondria and RNA by nanotubes, microvesicles, and exosomes (142). Specific characteristics and properties of MSCs may vary depending on their origin, variability in MSC donor types, production procedures and dose, as well as variations in study design, thus comparing different products can be demanding as specifically reviewed by Kelly and Rasko in 2021 (143). From the first treatment of a 9-year-old patient with SR aGvHD, achieving CR, reported in 2004 by Le Blanc, numerous studies and clinical trials have been conducted to investigate MSCs as a treatment for GvHD and most included patients with SR-aGvHD (134, 144). In 2008 an EBMT multicenter non-randomized study evaluated MSCs from either HLA-identical, haploidentical or unrelated HLA-mismatched donors in which 25 patients were children, who were found to respond consistently better than adults, with OR 80% vs. 60% in adults (*p* = 0.28) (136). In 2013 a retrospective analysis of 37 children with grade III-IV SR aGvHD treated with allogeneic MSCs CR of 65% and ORR 84% were reported. Patients with CR after MSC therapy had a cumulative incidence of transplant-related mortality of 17% compared to 69% unresponsive to MSCs (*p* = 0.001) (138). A multicenter, randomized, phase III clinical trial assessed the use of an industrial MSC product (remestemcel-L, Prochymal) in 260 patients (145), proving safety and tolerability but failing primary clinical endpoint of durable complete response of at least 28 days after beginning treatment in the intent-to-treat population, namely 35% vs. 30% (*p* = 0.42). Notably, a subset analysis of pediatric patients showed a higher ORR vs. placebo, namely 64% vs. 23% (*p* = 0.05) [55]. In 2021, an update on 241 pediatric patients with severe SR aGvHD was reported by Kurtzberg et al. Patients received biweekly infusions of 2 million MSCs/kg for four weeks, consistent with the schedule of the previous remestemcel-L trial. A total of 156 patients (65%) presented OR, with 34 (14.1%) achieving CR and 123 (51.3%) achieving PR. Survival through day 100 was 66.9% and was significantly higher in patients with OR on Day 28 than in non-responders, namely 82% vs. 39% (*p* < 0.001). Infusions were well tolerated, without evidence of infusion-related toxicities or ectopic tissue formation (146). The most frequent severe adverse events were infections, 24% of patients, and respiratory disorders in 16%. Subsequently, in 2021 a phase III, prospective, single-arm, multicenter study in 54 children with primary SR aGvHD was established with OR of 70%. Based on the available evidence, an attempt to obtain FDA approval was submitted to treat children with SR aGvHD with remestemcel-L, including a whole analysis of 309 children with GvHD who received remestemcel-L., but the application was declined, as a specific randomized controlled trial have been requested (146, 147). A limited number of studies have been conducted in cGvHD in adults, with variable OR reported, from 0 to 80%. Pediatric evidence is even scarcer. In 2008 Muller et al. reported 3 patients receiving MSC for extensive cGvHD with partial response in 1 (135). In the work of Lucchini et al. 5 cGvHD patients were included with 1 CR with flare, and 2 PR. Interestingly, *in vivo* immunomodulation was detected in responsive group (137) (Table 4).

### Fecal microbiota transplatation and microbial therapeutics

Gut microbiota composition has been linked to major complications in allogeneic allo-HSCT recipients (148–150). In particular, the relative abundance of specific bacterial taxa, such as Enterococcus expansion and reduction in Blautia, has been associated with aGvHD severity (151, 152). Based on this knowledge, various strategies have been developed to modulate the gut microbiota towards a protective configuration, ranging from antibiotic stewardship to nutritional modulation (153–155). Fecal microbiota transplantation (FMT) consists of the infusion of fecal microbiota from a healthy donor and has been proposed to directly restore the altered microbial composition observed in SR GI GvHD (156). In adults, encouraging preliminary data regarding feasibility and efficacy have been published, but larger prospective studies are lacking (157). To date, the use of FMT for steroid-refractory gut aGvHD in children has been reported in two 5-year-old patients. The first description was provided by Zhong et al. in 2019. FMT was performed twice on days +75 and +77 after HSCT via a nasojejunal tube from an unrelated donor and resulted in symptom remission without adverse events. Another case was described by Merli et al. in 2022. The child received FMT from his mother through upper GI endoscopy at a dose of 12 ml/kg on day +78 after HSCT after multiple lines of therapy, reaching complete remission. However, 20 days later the patient experienced gut aGvHD recurrence and underwent a second FMT from the same donor together with Begelomab, slowly reaching again remission of symptoms. About six months later, the patient developed a new flare of intestinal GvHD. The patient did not respond to steroids and mycophenolate and required surgery. Due to the persistence of symptoms, two other FMT infusions were performed from the uncle because of mother's unavailability, without clinical response. The patient then received Ustekinumab, achieving complete remission. At 5 years of follow-up, he was alive and did not present any signs of chronic GVHD, with normal intestinal function (158) (Table 4).

## Discussion

Treatment of SR-GvHD still represents a challenge in pediatric HSCT, particularly for very high-risk groups of severe GI-aGvHD and lung cGvHD/BOS. It is difficult to recommend a linear approach, since for long time most of the available evidence was assumed by retrospective experience, and more recent prospective study are limited by small numbers (4, 139). However, some considerations about indications can be outlined. Conventional drugs are generally affected by wider range of organ toxicity than other classes, especially in aGvHD setting. Nevertheless, an acceptable balance between efficacy and side effects has been reported in low-dose MTX for cGvHD (15, 20), and in MMF and pentostatin for aGvHD (16). Moreover, these drugs are usually easily accessible and manageable for clinicians and since few years ago represented the only choice for SR patients.

Mostly in the last 15 years, the interest to evaluate anti-cytokine therapies has grown, particularly in severe and GI aGvHD. Treatments were often translated from inflammatory bowel disease and autoimmune diseases. Even if a certain grade of activity was documented in terms of response, increase of infection rate was reported by most of the studies (81, 83, 95, 114). Overall survival in treated patients was characterized by lower trend than those observed in other classes of drugs. By a physio pathological point of view, it is worth noting that the inhibition of TNF*α* or other cytokines involved in GvHD does not directly affect T-cells, therefore GvHD is not eradicated. A similar phenomenon occurs in the other autoimmune diseases such as Chron's or rheumatoid arthritis. In this regard, all studies involving anti-cytokines report subsequent development of chronic GvHD in a non-neglectable percentage of patients.

Recently, introduction of targeted therapies was revolutionary for SR GvHD, reaching the lower rate of adverse events and probably the best efficacy currently available. Indeed, the advent of ruxolitinib has changed the landscape of treatment of SR acute and chronic GvHD. Pediatric experience flourished, reporting efficacy and low rate of treatment toxicity, with rare cases of treatment discontinuation (34–57). Results of the pivotal trial REACH4 (NCT03491215) will define if ruxolitinib use is destined to be introduced earlier in clinical practice, thus defining a novel concept of refractory GvHD. Furthermore, cGvHD setting has been renewed by introduction of ibrutinib. Brilliant response rates counterbalanced by a remarkably low burden of toxicity lead ibrutinib to be the first pediatric FDA-approved molecule for SR cGvHD. Promising results may also be obtained in patients with lung GvHD/BOS.

Finally, the class of non-pharmacological treatments comprise different approaches that share promising activity with relatively low toxicity but less feasibility than other therapies. ECP has been introduced since longer time in clinical practice, even if evidences in pediatric patients have been supported by only few retrospective studies (132, 162). Worst response was reported in severe aGvHD. Combination with ruxolitinib may be promising and allow to a higher rate of complete responses (139, 141). Use of MSCs has widespread in recent years (142, 143). Requirement of Good Manufacturing Practices (GMP) fulfillment for production represents a limitation for the diffusion of this approach, partially overcome by introduction of the industrial product remestemcel-L. The large prospective studies about remestemcel-L reported a low rate of adverse events with good responses, particularly in severe GI GVHD, but did not still obtain approval (146). Non-pharmacological treatments are attractive, considering the low toxicity rate due to broad immune-modulating effects rather than immune-suppressor activity. Combination or sequential use of different approaches may represent a promising tool to reach efficacious synergy and minimize side effects.

To summarize, we certainly achieved a wider range of possibilities for treatment of children with SR GvHD, even if a prevalent off-label use is currently available. In SR aGvHD, most pediatric clinicians nowadays recommend ruxolitinib as “standard” second-line therapy, similarly to the adult setting (159). Further addition of ECP or MSC can be supposed if response is unsatisfactory. Conventional and anti-cytokine therapies could also represent options, especially for gut GvHD, with careful attention to limit infections. In cGvHD, with the evidence available, both ruxolitinib and ibrutinib may be started after steroid refractoriness, conventional therapies as low dose MTX and imatinib can still represent good options, especially for lung involvement and BOS (20). ECP addition may be evaluated considering feasibility (132), and Belumosidil may be chosen in cases of sclerotic cGvHD (33).

Finally, clinical trials are currently ongoing for both SR aGvHD and cGvHD in children and adolescents. Beyond the previously mentioned pivotal trial REACH4 for aGvHD, three trials are evaluating cell therapies as MSCs (NCT04744116), decidua stroma cells (NCT04883918) and combination of MSCs with ruxolitinib in aGvHD (NCT04744116). Interestingly, a trial is assessing efficacy and safety of glucagon-like peptide-2 (GLP-2) apraglutide in gut SR aGvHD (NCT05415410). Among the most notable in refractory cGvHD, one trial is evaluating Treg enriched cell infusions (NCT05095649), and one is assessing hydrogen water, previously assessed in adult patients as a feasible and active approach with extreme safety (NCT02918188) (Table 5).

**Table 5:** Interventional trials in pediatric patients with steroid refractory aGvHD or cGvHD.

NCT Number	Study Status	Interventions	Sponsor	Phases	Setting
NCT04744116	Recruiting	Ruxolitinib + Mesenchymal Stem Cells	Academic	Phase I	Adolescent aGvHD
NCT04289103	Not yet recruiting	Inolimomab	Company	Phase III	Pediatric aGvHD
NCT04629833	Recruiting	Mesenchymal Stem Cells	Company	Phase III	Adolescent aGvHD
NCT05095649	Recruiting	Regulatory T-cell enriched infusion	Academic	Phase II	Pediatric cGvHD
NCT04883918	Not yet recruiting	Decidua stromal cells	Company	Phase II	Pediatric aGvHD
NCT05415410	Recruiting	Apraglutide	Company	Phase II	Pediatric aGvHD
NCT02918188	Recruiting	Hydrogen	Academic	Phase II	Pediatric cGvHD

## Conclusions and future perspectives

Best management of SR-GvHD in pediatrics is still undetermined due to lack of prospective and randomized studies. Nevertheless, differently from the past, pediatric hematologists are now equipped with a growing number of therapeutic instruments. Management of SR aGvHD have been renewed by introduction of ruxolitinib, which demonstrated remarkable efficacy and safety, potentially reducing the rate of refractory patients, if used as first-line approach. Non pharmacologic treatments, particularly MSCs may be promising for SR aGvHD even in high-risk patients, as acting by modulating rather than suppressing immune system. Cost-benefit ratio due to effort of obtaining and performing a cell therapy in this setting may be less favorable and to be reserved to selected cases. Regarding cGvHD, both ruxolitinib and ibrutinib have transformed the landscape of this complication, demonstrating good efficacy and excellent safety. Also in this context, an early introduction in clinical practice may potentially change the paradigm of “refractoriness”. New approaches might represent in the future further lines in “ruxolitinib-refractory” and “ibrutinib-refractory” GVHD. Of note, different mechanisms of action are targeted by different treatments, and exploring combinations may exploit the efficacy. Perspective trials to compare different strategies should be supported and encouraged through centers.
